# A Hat-Pin in the Stomach

**Published:** 1900-01-13

**Authors:** 


### A HAT-PIN IN THE STOMACH.

A very curious and interesting case is recorded by Mr. John Halliwell,[1] of Winchcombe, in which a hat-pin 4½ inches long was removed from the stomach of a woman, aged 60, four days after it had been swallowed. The patient had been eating a crust of bread when it stuck in her throat. She endeavoured to force it down with her fingers but failed, and, feeling herself choking, she passed a bonnet-pin down head foremost to dislodge it. In doing this it is to be presumed she was successful, but the point of the pin stuck fast in her palate, and while disengaging it from this entanglement it slipped from her fingers and was swallowed. She felt little of it for two days, when she began to feel a pricking in the epigastric region. On examination nothing could be felt, but on deep pressure over the left iliac fossa she complained of sharp pricking pains, which she also felt when she moved, coughed, or stooped. The pain had quite left the epigastric region; a point of interest, seeing that the pin was still in the stomach. On the fourth day after the accident laparotomy was performed, and the pin was found to be in the stomach, the head lying upwards and to the right, and the point downwards and to the left. The point was imbedded, and could not be extricated by manipulation, so, instead of incising the stomach, the point was forced through the hole which was already made by its point, and being then drawn out as far as it would come, was cut off with a pair of bone forceps close to the head, the latter being left to be passed *per vias naturales*, which happened about 10 days afterwards. She made a good recovery.

[1] Lancet, Dec. 23.

